# Peripheral Blood-Based Biomarkers for Immune Checkpoint Inhibitors

**DOI:** 10.3390/ijms22179414

**Published:** 2021-08-30

**Authors:** Ho Jung An, Hong Jae Chon, Chan Kim

**Affiliations:** 1 Department of Medical Oncology, St. Vincent’s Hospital, College of Medicine, The Catholic University of Korea, Seoul 06591, Korea; meicy@catholic.ac.kr; 2 Medical Oncology, CHA Bundang Medical Center, CHA University School of Medicine, Seongnam 13496, Korea

**Keywords:** peripheral blood, biomarker, cancer immunotherapy, immune checkpoint inhibitor

## Abstract

As cancer immunotherapy using immune checkpoint inhibitors (ICIs) is rapidly evolving in clinical practice, it is necessary to identify biomarkers that will allow the selection of cancer patients who will benefit most or least from ICIs and to longitudinally monitor patients’ immune responses during treatment. Various peripheral blood-based immune biomarkers are being identified with recent advances in high-throughput multiplexed analytical technologies. The identification of these biomarkers, which can be easily detected in blood samples using non-invasive and repeatable methods, will contribute to overcoming the limitations of previously used tissue-based biomarkers. Here, we discuss the potential of circulating immune cells, soluble immune and inflammatory molecules, circulating tumor cells and DNA, exosomes, and the blood-based tumor mutational burden, as biomarkers for the prediction of immune responses and clinical benefit from ICI treatment in patients with advanced cancer.

## 1. Introduction

In the past decade, cancer immunotherapy using immune checkpoint inhibitors (ICIs) has demonstrated promising clinical efficacy in the treatment of various malignancies. It enabled the complete regression of advanced tumors, resulting in long-term survival in a fraction of patients [1,2,3,4]. However, this potent immunotherapeutic efficacy is not always possible due to an immunosuppressive tumor microenvironment (TME) that lacks or excludes anti-tumor effector T cells [5,6,7,8,9]. Therefore, the identification of patients who are likely or unlikely to respond to ICI treatment before or as early as possible during treatment is crucial to elicit optimal immunotherapeutic efficacy [2,10,11].

Tumor programmed death-ligand1 (PD-L1) expression was initially suggested as a plausible biomarker for predicting the response to anti-PD-1/PD-L1 therapy. A multitude of clinical studies have demonstrated the enhanced efficacy of immune checkpoint blockade in patients with higher levels of intratumoral PD-L1 [2,4,11,12]. However, tumoral PD-L1 positivity alone is insufficient for patient stratification because some PD-L1 negative patients also respond to immunotherapy and PD-L1 levels show spatiotemporal variations during cancer treatment [10,13]. Other tumor factors associated with enhanced clinical benefit from ICIs include mismatch repair protein deficiency, microsatellite instability (MSI), high tumor mutational burden (TMB), and the effector T-cell gene signature [2,4,10,12,14].

Although tumor tissue-based biomarkers partially aid in identifying patients who will benefit more from ICIs, many challenges still exist in clinical practice [4,11,12,15,16,17]. First, tumor biopsies are generally invasive, and obtaining patient tissue samples using biopsy is severely limited by tumor accessibility and the condition of the patient. Furthermore, repeated tissue biopsy could increase the likelihood of procedure-related complications and delay cancer treatment. Moreover, because of tumor heterogeneity, the local immune response within one metastatic lesion may not represent the systemic anti-cancer immunity of the patient. Lastly, although cancer immunity may constantly change during ICI treatment, longitudinal immune monitoring using repeated tissue biopsy is usually not feasible in the clinic, especially during rapid clinical deterioration. Therefore, clinicians usually make decisions based on a singe-timepoint tumor biopsy at the time of treatment initiation, rather than performing repeated tumor biopsies to monitor the updated immunological profiles of the TME.

**Figure 1:**
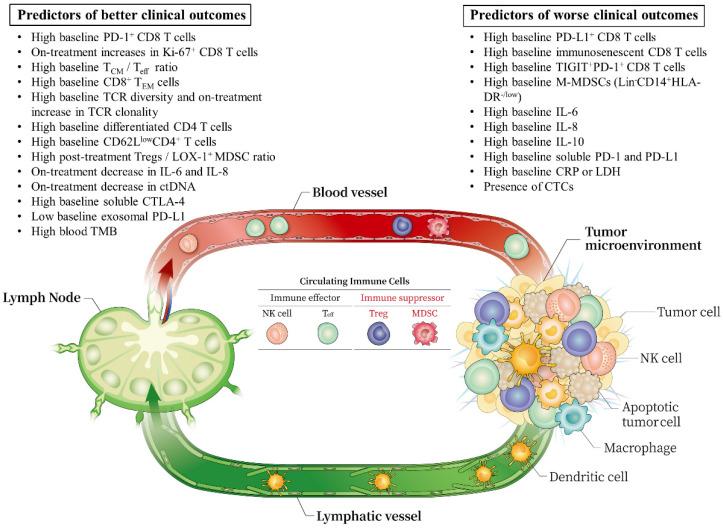
Summary of various circulating immune biomarkers during ICI treatment. Central memory T cells, TCM; effector memory T cells, TEM; effector T cells, Teff; T cell receptor, TCR; regulatory T cells, Tregs; myeloid-derived suppressor cells, MDSCs; polymorphonuclear-MDSCs, PMN-MDSCs; tumor mutation burden, TMB; circulating tumor cells, CTCs; circulating tumor DNA, ctDNA; monocytic-MDSCs, M-MDSCs; natural killer cell, NK cell.

With the advent of high-throughput multiplexed analytical technologies, peripheral blood is now suitable for deeper immune profiling. As peripheral blood sampling is readily available, minimally invasive, and repeatable, the use of blood-based immune biomarkers can compensate for the abovementioned limitations of tissue-based immune biomarkers during cancer immunotherapy [11,15,18,19,20,21,22,23]. This review aimed to summarize pivotal findings related to blood-based immune biomarkers in patients with solid cancer treated with ICIs (Figure 1).

## 2. Circulating Immune Cells

There are various immune cell components circulating in peripheral blood including T cells, B cells, natural killer (NK) cells, or myeloid cells. As circulating immune cells could reflect systemic immune responses, they can help predict therapeutic responses and clinical benefit of ICIs in patients with advanced cancers [23,24,25]. Recent technical advances in the detection of various immune cell subsets using multi-color fluorescence flow cytometry, mass cytometry, and next generation sequencing have enabled the identification and monitoring of different circulating immune cell subtypes in peripheral blood [11,14,15,18,22,23].

Table 1 summarizes the circulating immune cell biomarkers used for predicting the clinical benefit from various ICI treatments. Many studies have analyzed in detail various immune cell subpopulations to distinguish their functional phenotypes and differentiation status because anti-tumor immunity responses are very complex processes and have analyzed the dynamics of each cell population at various time points before and after ICI treatment.

### 2.1. CD8 T Cells

CD8 T cells are known as the most potent cytotoxic effectors against non-self antigens, including cancer, and are the major targets of current cancer immunotherapies [5,46,47,48]. Circulating CD8 T cells have been explored, among immune cells, as potential determinants of the clinical benefit from ICI therapy.

In a study of patients with non-small cell lung cancer (NSCLC) receiving PD-(L)1 inhibitor treatment, fewer circulating CD8 T cells prior to ICI was associated with durable clinical benefit (DCB) (accuracy = 70%) [18]. The decrease in the number of circulating T cells in the peripheral blood might be due to the homing of most T cells into the tumor tissue. The authors also generated a predictive model combining non-invasive parameters, including baseline circulating CD8 T cells, blood-based tumor mutational burden, and early changes in circulating tumor DNA (ctDNA) that could more accurately predict DCB (accuracy = 92%) and longer median progression-free survival (PFS) following ICI treatment.

Although the total number of circulating CD8 T cells in peripheral blood may reflect the overall immunologic status, the T cell subtypes involved in cancer immunity are very heterogeneous, and many of them are not tumor-specific [49,50]. Therefore, in most other studies, investigators have performed a more detailed analysis of T cell subtypes, rather than measuring the total T cell number.

### 2.2. PD-1 or PD-L1 Expressing CD8 T Cells

The interaction between PD-1 and PD-L1 governs T cell immune tolerance and exhaustion during the cancer-immunity cycle. PD-1-overexpressing circulating T cells are more frequently detected in cancer patients than in healthy individuals [51,52]. Intriguingly, tumor neoantigen-specific T cells can be found in circulating PD-1^+^ CD8 T cells, but not in PD-1^−^ CD8 T cells. Therefore, PD-1^+^ CD8 T cells in the peripheral blood could be used as a non-invasive surrogate to monitor neoantigen-reactive T cells that reside within the tumor [50,53].

In patients with unresectable stage III or IV melanoma treated with ipilimumab, higher pretreatment levels of PD-L1^+^ CD8 T cells in peripheral blood were correlated with poor overall survival (OS), while showing a marginal association with ipilimumab response [26].

Meanwhile, in patients with NSCLC who were pretreated with nivolumab, PD-1^+^ CD8 T cells at baseline were more frequently detected in patients with clinical benefit (CB) than in those with non-response (NR) [27]. Furthermore, a recent study showed that a higher percentage of circulating PD-1^+^ CD8 T cells at baseline was associated with a clinical benefit in patients with MSI-high gastric cancer treated with pembrolizumab [28].

Early dynamic changes in PD-1^+^ CD8 T cells have also demonstrated consistent results in predicting the response to ICIs. As PD-1/L1 blockade could reinvigorate PD-1-expressing CD8 T cells and induce their activation and proliferation, early proliferation of circulating PD-1^+^ CD8 T cells following ICI treatment has been investigated in several studies [29,30,31]. Kamphorst et al. observed an increase in Ki-67^+^ PD-1^+^ CD8 T cells in the peripheral blood of NSCLC patients treated with PD-1 inhibitors. These proliferative T cells showed an effector phenotype with upregulated costimulatory molecules and high levels of PD-1 and cytotoxic T-lymphocyte-associated protein (CTLA-4). Patients with early proliferative T cell responses within 4 weeks of treatment showed CB from PD-1 targeted therapy, while those with delayed or absent responses usually experienced disease progression [29]. Similarly, an increase in Ki-67^+^ PD-1^+^ CD8 T cells after PD-1 inhibitor treatment was also reported in patients with advanced melanoma treated with pembrolizumab [30]. Intriguingly, these responding peripheral blood T cells were mostly PD-1^+^, predominantly had an exhausted phenotype (T_ex_), and shared T cell receptor (TCR) clonotypes with tumor-infiltrating T cells. Moreover, a ratio of Ki-67^+^ CD8 T cells to tumor burden >1.94 at week 6 was correlated with a better response rate (RR), and longer PFS and OS. Another study supported these findings prospectively in thymic epithelial tumors (TETs) and NSCLC [31]. The authors analyzed the change in Ki-67^+^ cells among PD-1^+^ CD8 T cells in TET patients treated with pembrolizumab and identified early proliferation of PD-1^+^ CD8 T cells one week after the initiation of anti-PD1 therapies. When a fold change in Ki-67^+^ PD-1^+^ CD8 T cells at baseline and 7 days after ICI treatment (Ki-67 _D7/D0_) was observed, Ki-67 _D7/D0_ was significantly higher in patients with DCB than in those without DCB. An optimal cut-off of Ki-67 _D7/D0_ ≥2.8 was determined in the TET and NSCLC cohort, which was associated with higher DCB, and longer PFS, or OS. In a validation cohort of NSCLC patients treated with PD1 inhibitors, Ki-67 _D7/D0_ consistently predicted better DCB, PFS, and OS. Taken together, early proliferation of circulating PD-1^+^ CD8 T cells after ICI treatment showed consistent potential as a predictive biomarker for favorable clinical response and outcomes. Further studies are warranted to confirm which time point would be the best for detecting dynamic changes in PD-1^+^ CD8 T cells. 

### 2.3. TIM-3 Expressing T Cells

In addition to PD-1, other immune checkpoint molecules have been investigated. When paired blood samples were analyzed at baseline and at least at 8 weeks after treatment initiation with PD1 inhibitors in NSCLC and renal cell carcinoma, the frequency of TIM-3-expressing CD4 or CD8 T cells was significantly increased in patients with progressive disease (PD) compared to that of patients without PD [32]. Moreover, increases in TIM-3^+^ cells in either CD4 or CD8 T cells were negatively correlated with PFS. However, opposite results were observed in patients with esophageal squamous cell carcinoma. In esophageal cancer, an increase in TIM-3-expressing CD4 or CD8 T cells after the first cycle of nivolumab treatment was associated with higher clinical responses or longer OS [33]. These controversial results should be validated in future studies with larger sample sizes and robust longitudinal analyses at various time points.

### 2.4. Immunosenescent CD8 T Cells

Immunosenescence is a gradual deterioration of the immune system due to aging processes and is associated with an increased susceptibility to infection and malignancy [54]. Senescent T cells display a terminal differentiated phenotype with low proliferative activity and reduced TCR diversity pool, but still have a cytotoxic potential, which is distinguishable from that of exhausted T cells [55]. The loss of CD28, increased expression of CD57 or killer-cell lectin-like receptor (KLRG1) expression were used as T cell immunosenescence markers [56]. In NSCLC patients treated with PD-(L)1 inhibitors, the pretreatment percentage of CD28^−^ CD57^+^ KLRG1^+^ cells among CD8 T cells, defined as the senescent immune phenotype (SIP), was correlated with poor outcomes [34]. A cut-off of 39.5% of SIP among CD8 T cells could predict poor clinical benefit from PD-(L)1 blockade (sensitivity 100%, specificity 35%). Using this cut-off, patients with SIP showed lower RR and worse PFS or OS to PD-(L)1 inhibitors compared to those of patients without SIP, while these findings were not observed in a separate cohort treated with conventional chemotherapy. Furthermore, compared to patients without SIP, patients with SIP were more frequent affected by hyperprogressive disease (HPD). SIP^+^ CD8 T cells were terminally differentiated T cells with a low proliferation index and reduced secretion of pro-inflammatory cytokines. These results suggest that circulating T cells with an immunosenescence phenotype could be a potential predictive biomarker of ICI treatment outcomes.

### 2.5. Memory T Cells

T cells undergo a natural differentiation since antigen recognition and are phenotypically subdivided based on the following surface markers: naïve T cells (T_N_, CD45 RO^−^ CCR7^+^), central memory T cells (T_CM_, CD45 RO^+^ CCR7^+^), effector memory T cells (T_EM_, CD45 RO^+^ CCR7 ^−^), and terminal effector T cells (T_TE_, CD45 RO^−^ CCR7^−^) [57,58]. T cells at each stage of differentiation could have a distinct role in ICI-induced restoration of anti-tumor immunity and show varied potential as predictive biomarkers of ICI treatment outcomes. It seems that a higher frequency of memory T cells compared to that of effector T cells (T_eff_) in the peripheral blood at baseline were associated with better response and prognosis after ICI treatment [35,58]. However, this finding needs further validation in prospective studies due to the heterogeneous population studied and inconsistent sample processing.

Some researchers have reported the predictive potential of T_CM_ cells in ICI treatment response. Circulating CD4^+^ T_CM_ cells seem to be associated with a good response and better prognosis in NSCLC and RCC patients receiving anti-PD-1 inhibitors [32]. Furthermore, Manjarrez-Orduno et al. found that patients whose tumors exhibited increased inflamed signature and PD-L1 expression showed higher levels of CD4^+^ and CD8^+^ T_CM_ cells compared to those of T_eff_ cells in the peripheral blood of patients with melanoma and NSCLC [35]. Intriguingly, a high T_CM_/T_eff_ ratio at baseline was associated with longer PFS (*p* < 0.05) in a NSCLC cohort treated with nivolumab. However, there were no major changes in the T_CM_/T_eff_ ratio in the follow-up blood samples at 3 months. 

Other studies have revealed the potential role of T_EM_ cells in predicting the clinical benefits of ICIs. In patients with melanoma treated with PD-1 inhibitors, a higher frequency of circulating CD8^+^ T_EM_ cells and lower frequency of CD4^+^ T_EM_ cells and naïve CD8^+^ T cells at baseline were observed in responders [36]. Consistent results were also reported in melanoma patients treated with the anti-CTLA-4 antibody, ipilimumab. Wistuba-Hamprecht et al. demonstrated that high frequencies of CD8 effector-memory type 1 T cells defined as CD45 RA^−^ CCR7^−^ CD27^+^ CD28^+^ in the peripheral blood within 4 weeks of treatment initiation were associated with higher clinical response and longer OS [37]. However, late stage-differentiated effector memory CD8 cells (CD45 RA^+^ CDR7^−^CD27^−^CD28^−^) were inversely related to OS. Furthermore, other researchers have simultaneously investigated the functional status and phenotype. Kim et al. reported that a lower frequency of CD8^+^ T_EM_ cells and a higher frequency of severely exhausted T cells (TIGIT^+^ cells among PD-1^+^ CD8^+^ T cells) at baseline were associated with HPD and shorter OS in NSCLC patients treated with PD-(L)1 inhibitors [38].

Overall, memory T cells in the peripheral blood at a certain differentiation stage could be considered when predicting responses to ICIs.

### 2.6. TCR Clonality and Diversity of PD-1^+^ CD8^+^ T Cells

Some researchers have focused on the role of TCR clonality in circulating T cells as a predictive determinant of ICI response. In NSCLC, dominant TCR expansion was observed not only within the tumor tissue but also in circulating T cells, and early and sustained TCR clonal expansions in the blood were present in ICI responders [56,59] Recently, Han et al. reported that pretreatment PD-1^+^ CD8^+^ TCR diversity in the peripheral blood was higher in patients with disease control than in those with disease progression, and high PD-1^+^ CD8^+^ TCR diversity (>3.14) was associated with better response, longer PFS, and OS in NSCLC patients treated with PD-(L)1 inhibitors [39]. Increasing PD-1^+^ CD8^+^ TCR clonality at 4–6 weeks after ICI treatment initiation was associated with a higher disease control rate, longer PFS, and OS. Moreover, TCR diversity was an independent prognostic factor for both PFS and OS. The higher TCR diversity of PD-1^+^ CD8 T cells might reflect a higher probability of neoantigen recognition. After ICI exposure, the dominant clonal expansion of tumor-specific T cells indicated a good clinical response. Furthermore, it differentiated pseudo-PD from true PD. Thus, monitoring the TCR repertoire could help predict the ICI benefit. Expansion of TCR clones in the peripheral blood after ICI treatment may not completely reflect the true diversity of the TCR repertoire within the tumor and may not be sufficient to suppress tumor growth. A recent study by El Meskini et al. elucidated this by using genetically engineered mouse melanoma models. They demonstrated that the tumor response to ICI therapy requires not only clonal expansion of the TCR repertoire but also tumor access to adequate TCRs [60]. Therefore, the post-treatment expansion of TCR clones in both the blood and tumor seems critical for the therapeutic response to ICIs. The dynamic changes in TCR clonality and diversity during ICI treatment could be validated in future prospective studies.

### 2.7. CD4 T Cells

CD4 T cells can promote anti-tumor immunity by supporting the priming, migration, and survival of CD8 T cells [61]. Moreover, functional CD4^+^ T cells are necessary to restore the cytotoxicity of CD8 T cells following anti-PD-(L)1 treatment. In NSCLC patients treated with PD(L)1 inhibitors, a high proportion (>40%) of highly differentiated CD4 T cells (CD27^−^CD28 ^low/negative^) in peripheral blood at baseline could predict objective RR with 100% specificity and 70% sensitivity [40]. Furthermore, this was correlated with longer PFS in multivariate analysis. These CD4 T cells mainly expressed memory features with higher Ki67^+^ expression, but lower co-expression of PD-1 and LAG-3. Moreover, a low percentage of CD25^+^ FOXP3^+^ CD4^+^ Tregs was associated with a higher RR and longer PFS and OS.

A recent report showed that CD62 L^low^ CD4 T cells were associated with the clinical response to nivolumab in NSCLC [41]. The circulating level of CD62 L^low^ CD4 T cells at baseline was higher in the nivolumab responders compared to that in non-responders (*p* < 0.001). These cells showed the classical characteristics of Th1 cells and were positively correlated with the percentage of effector CD8 T cells and expression of PD-1 on CD8 T cells. Conversely, the percentage of CD25^+^ FOXP3^+^ CD4^+^ Tregs was significantly lower in nivolumab responders compared to that in non-responders. Intriguingly, durable responders maintained high percentages of CD62 L^low^ CD4 T cells within the peripheral blood. 

Taken together, the intrinsic functionality of CD4 T cell immunity could be a key factor for restoring anti-tumor immunity and predicting outcomes in patients treated with ICIs.

### 2.8. Immunosuppressive Cells: Myeloid-Derived Suppressive Cells (MDSCs) and Tregs

MDSCs are a heterogeneous group of myeloid cells that fail to differentiate into granulocytes, macrophages, or dendritic cells. They show immunosuppressive activity by inhibiting T and NK cells, stimulating Tregs, and playing an important role in various malignancies [5,62]. MDSCs are classified into two phenotypes: neutrophil-like MDSCs, called granulocytic-MDSCs (G-MDSCs), also known as polymorphonuclear-MDSCs (PMN-MDSCs), and monocyte-like MDSCs (M-MDSCs). G-MDSCs are commonly identified as Lin^−^ CD11b^+^ CD14^−^ CD15^+^ HLA-DR^−^ or Lin^−^ CD11b^+^ CD14^−^ CD66b^+^, and M-MDSCs are defined as Lin^−^ CD11b^+^ CD14^+^ CD15^−^ HLA-DR^−/low^. Along with MDSCs, Tregs (CD4^+^ CD25^+^ FoxP3^+^) play another key immunosuppressive role in various underlying mechanisms [5,63,64]. They are a subset of CD4 T cells that maintain immune homeostasis by hindering the activities of CD4 and CD8 effector cells, NK cells, and antigen-presenting cells [5,64]. Therefore, these immunosuppressive cells have been investigated as potential prognostic or predictive biomarkers in cancer patients receiving ICIs. 

A study examined circulating M-MDSCs (Lin^−^ CD14^+^ CD11b^+^ HLA-DR^low/−^) in melanoma patients receiving ipilimumab [42]. M-MDSCs at baseline were more frequent in melanoma patients than in healthy volunteers. With a cut-off of 14.9% of M-MDSCs, patients with fewer M-MDSCs at baseline or at week 6 survived longer than patients with more abundant M-MDSCs. There was a statistically significant inverse correlation between percentage changes in the CD8^+^ T cell number and M-MDSC frequency at week 6.

Martens et al. analyzed M-MDSCs and Tregs in a large cohort of patients with advanced melanoma treated with ipilimumab [43]. This study revealed that M-MDSCs (Lin^−^CD14^+^ HLA-DR^−/low^) at baseline were negatively correlated with OS, while Tregs were positively correlated with OS. When these two parameters, M-MDSCs ≥ 5.1% and Tregs < 1.5%, were integrated with other clinical parameters into a prognostic model, OS and ipilimumab responses could be better predicted. 

Furthermore, another study in patients with advanced melanoma treated with ipilimumab as a neoadjuvant, investigated the early dynamic changes in circulating MDSCs and Tregs at week 6. It was found that early on-treatment decrease in M-MDSCs (Lin1^−^HLA-DR^−^ CD33^+^ CD11b^+^) and an increase in Tregs at week 6 were associated with longer PFS [44].

As MDSCs share common features with neutrophils, it is challenging to clearly distinguish MDSCs from neutrophils. Recently, lectin-type oxidized LDL receptor-1 (LOX-1) has been suggested as an MDSC-specific marker in patients with cancer [65]. A recent study examined LOX-1-expressing PMN-MDSCs and Tregs in NSCLC patients treated with nivolumab [45]. In the exploratory cohort, the baseline level of LOX-1^+^ PMN-MDSCs in the peripheral blood did not differ between responders and non-responders, while the level of Tregs was higher in responders compared to that in non-responders. Interestingly, LOX-1^+^ PMN-MDSCs were significantly decreased in responders after nivolumab treatment, but the levels of Tregs were unaltered. There was an inverse correlation between LOX-1^+^ PMN-MDSCs and Tregs, and a ratio of Tregs to LOX-1^+^ PMN-MDSCs (TMR) ≥ 0.39 could distinguish the nivolumab responders from the non-responders. A high post-treatment TMR was also significantly correlated with longer PFS. Therefore, relative changes in Tregs and PMN-MDSC during PD-1 inhibition could help predict a good response to and better prognosis from ICI treatment.

Taken together, MDSCs were consistently associated with poor ICI responses and clinical outcomes. However, regarding Tregs, the results are controversial; while some studies revealed a positive correlation, another study reported a negative correlation, and other studies reported no such relationship between Tregs and ICI outcomes [27,30,36,41,43,45]. Therefore, the clinical implication of Tregs in cancer patients treated with ICIs requires further investigation.

### 2.9. Natural Killer (NK) Cells

NK cells induce adaptive immune responses without prior antigen sensitization during ICI treatment [66]. Mazzaschi et al. showed that the NSCLC patient group that benefited from nivolumab treatment showed higher CD56^+^ NK cells with a more cytotoxic phenotype (perforin, granzyme B, CD3ζ) than the non-responding group [27]. High baseline levels of circulating NK cells (>202/µL) were associated with prolonged OS in this study. A recent study reported similar results in NSCLC [67], which require further validation due to the small sample size.

## 3. Cytokines and Soluble Proteins

Cytokines are immunological signaling proteins that are released into the systemic circulation and act primarily at the local cellular level [11,68]. Across malignancies, the changes of cytokine gene expression are accompanied by responses to ICIs. These immunological changes precede changes in the tumor burden in imaging studies and serve as a reflection of the ICI mechanism. Many of these changes are associated with soluble proteins, cytokines, and chemokines that can enter the systemic circulation [11,15,69,70,71]. Various reports have demonstrated the use of soluble factors, such as IL-6, IL-8, soluble CTLA-4 (sCTLA-4), soluble PD-1 (sPD-1), soluble PD-L1 (sPD-L1), CRP, and LDH, as either predictive or prognostic factors for immunotherapy response (Table 2). These cytokines and soluble checkpoint molecules can easily be measured using ELISA, facilitating automated, highly sensitive, and accurate analyses of multiple peripheral blood samples.

### 3.1. IL-6

IL-6 is secreted by various cell types, including immune and tumor cells. It promotes tumor progression by inhibiting cancer cell apoptosis and promoting tumor angiogenesis [88]. In a previous study, higher serum IL-6 levels were associated with shorter OS in patients with advanced melanoma receiving IL-2 immunotherapy [89]. On the other hand, decreased IL-6 levels after anti-PD-(L)1 therapy were associated with improved PFS and correlated with changes in CRP levels in patients with NSCLC [70].

### 3.2. IL-8

IL-8 is a member of the CXC chemokine family, which is important for neutrophil chemotaxis [71,90]. IL-8 is secreted by tumor cells and tumor stroma cells in multiple tumor types. Multiple mechanisms are associated with the protumoral activities of IL-8, some of which directly affect tumor endothelial cells and cancer stem cells, and indirectly attract and modulate tumor-associated myeloid cells [91,92]. Sanmamed et al. reported that early on-treatment decreases in serum IL-8 levels were associated with longer OS in patients with melanoma and NSCLC receiving anti-PD-1-based therapy [72]. Furthermore, they showed that serum IL-8 levels were correlated with tumoral IL-8 production. Moreover, serum IL-8 levels could correctly distinguish radiologic pseudoprogression from real disease progression. Another study demonstrated the predictive role of IL-8 in 1445 patients with urothelial carcinoma (UC) and RCC treated with atezolizumab in clinical trials (IMvigor210, IMvigor211, and IMmotion 150) [71]. In this study, high baseline levels of plasma IL-8 were significantly associated with decreased efficacy and poor prognosis with atezolizumab treatment in UC and RCC. On-treatment decreases in plasma IL-8 levels were correlated with improved OS in patients with UC. Single-cell RNA sequencing analyses also revealed that myeloid cells in peripheral blood and the TME mainly expressed IL-8, and high expression of IL-8 was correlated with the downregulation of antigen presentation.

### 3.3. IL-10

IL-10 plays a pleomorphic role in cancer immunity [93]. It has been classically considered an immunosuppressive factor that promotes tumor growth, while recent studies showed exogenous IL-10 administration can induce anti-tumor effects by promoting CD8 T cell-mediated anti-tumor immunity [94,95]. High levels of serum IL-10 correlated with a worse survival outcome in both solid and hematologic malignancies [96]. In patients with locoregionally advanced melanoma treated with neoadjuvant ipilimumab, a high baseline IL-10 level appeared a biomarker for progression, and high TGF-β levels for non-progression. The combination of high IL-10 and low TGF-β at baseline was significantly correlated with worse PFS (HR 2.66, *p* = 0.035) [73]. Recently, Giunta et al. reported high baseline IFN-γ/IL-10 ratio in peripheral blood mononuclear cells could predict the clinical response and a longer PFS in patients with advanced melanoma treated with either nivolumab or pembrolizumab [74].

### 3.4. Soluble CTLA-4 (sCTLA-4)

Recent studies have attempted to elucidate the role of soluble checkpoint molecules in immune regulation. sCTLA-4 is primarily secreted by Tregs, although monocytes and immature DCs were also found to have sCTLA-4 transcripts. sCTLA-4 can be detected in normal human serum, but its levels are increased in many cancer types [97]. Pistill et al. demonstrated that melanoma patients with high serum levels of sCTLA-4 (>200 pg/mL) at baseline had better ORR and OS with ipilimumab treatment than those with lower sCTLA-4 serum levels (≤200 pg/mL), suggesting that serum sCTLA-4 could serve as a biomarker for predicting the response to ipilimumab [75]. Intriguingly, higher baseline sCTLA-4 levels were correlated with more frequent onset of immune-related adverse events, especially in those of the gastrointestinal tract.

### 3.5. Soluble PD-1 (sPD-1) or PD-L1 (sPD-L1)

Tumor cells and mature DCs produce and release soluble PD-L1 (sPD-L1) [98]. However, the potential use of sPD-L1 as a predictive biomarker remains to be determined. Ugurel et al. reported that melanoma patients had higher sPD-1 and PD-L1 levels than healthy controls [76]. They revealed that elevated sPD-1 and sPD-L1 levels at baseline correlated with poor responses to PD-1 blockade, but not to BRAF blockade in patients with metastatic melanoma. Moreover, patients with lower sPD-1 and sPD-L1 levels at baseline showed longer PFS and OS than those with higher sPD-1 and/or sPD-L1 levels. Consistently, other studies have also shown that higher baseline levels of sPD-1 or sPD-L1 were associated with poor clinical outcomes of ICI therapy in melanoma [99,100].

### 3.6. CRP

CRP is a serum inflammatory protein produced by hepatocytes. Its serum levels increase quickly in response to most types of inflammation, and elevated CRP levels have been reported to be associated with an increased risk of cancer [101]. High CRP levels at baseline were reported to be associated with poor PFS and OS in cancer patients treated with PD-1 inhibitors [77]. On the other hand, a recent study showed that the CRP flare-response, in which CRP rises rapidly at a very early phase of treatment and then falls shortly thereafter, is associated with better clinical outcomes in patients with RCC treated with nivolumab [78].

### 3.7. LDH

The baseline serum level of LDH is often regarded as an independent predictive fac-tor for shorter OS in patients with advanced melanoma treated with ipilimumab or pembrolizumab. Several studies have shown that high baseline serum LDH levels are associated with poor anti-tumor response in patients with various malignancies treated with immunotherapies [79,80].

## 4. Circulating Tumor Cells and Tumor Cell-Derived Factors

### 4.1. Circulating Tumor Cells (CTCs)

CTCs are cancer cells that detach from the tumor tissue and circulate freely in the bloodstream. Since CTCs are closely associated with the overall tumor burden, tumor invasiveness, and the likelihood of hematogenous metastasis, the presence of CTCs is an independent poor prognostic factor in various cancer types [102,103,104]. Therefore, CTC measurement using liquid biopsy is performed for cancer diagnosis, molecular profiling, and treatment response monitoring [102,104]. Tamminga et al. reported that CTCs were detected in one-third of patients with advanced NSCLC at baseline or at 4–6 weeks after ICI treatment initiation. They also showed that NSCLC patients with CTCs had worse PFS and OS than those without CTCs. Intriguingly, approximately 50% of patients with NSCLC without CTCs at both time points or with decreasing CTCs during ICI treatment exhibited a durable treatment response [81]. Therefore, analyzing the frequency and dynamics of CTCs, especially in NSCLC patients without available tumor tissue, can help predict ICI treatment response at an early time point.

### 4.2. ctDNA

DNA can be released from apoptotic or necrotic tumor cells into the systemic circulation. This tumor-derived circulating DNA, ctDNA, has a short half-life and can be utilized as a real-time biomarker for monitoring immunologic responses after ICI treatment [15,104,105]. In a retrospective study of patients with NSCLC and UC treated with the anti-PD-1 antibody, durvalumab, changes in ctDNA variant allele frequency (VAF) were correlated with clinical outcome (early reduction in ctDNA VAF at 6 weeks after treatment initiation was associated with longer PFS and OS) [82]. Another study also revealed that a decrease in ctDNA allele frequency between the baseline and first on-treatment sampling was correlated with higher RR, longer PFS (8.3 vs. 3.4 months, *p* = 0.0007), and longer OS (26.2 vs. 13.2 months, *p* = 0.008) in patients with advanced NSCLC treated with first-line pembrolizumab ± chemotherapy [83]. Finally, in a phase II clinical trial in which patients with advanced solid tumors were treated with pembrolizumab, the baseline concentration of ctDNA was strongly correlated with PFS, OS, and clinical responses. This predictive power was much stronger when the ctDNA kinetics during treatment were reflected. Intriguingly, all patients with complete ctDNA clearance remained alive during follow-up for more than 2 years [84]. Collectively, early ctDNA changes in patients treated with ICIs can be used as a potential biomarker to capture early immunological responses and survival outcomes.

### 4.3. Blood TMB

TMB is a surrogate marker for increased presence of tumor neoantigens and is thus used to predict the clinical benefit of anti-PD-1/PD-L1 therapy [15]. Wang et al. reported that blood TMB (bTMB) estimated using a cancer gene panel, NCC-GP150, was highly correlated with tissue TMB calculated using whole exome sequencing. In NSCLC patients treated with anti-PD or anti-PD-L1 antibodies, bTMB ≥ 6 was associated with higher response rates (39.3% vs. 9.1%, *p* = 0.02) and superior PFS (HR 0.39, *p* = 0.01) [106].

In another study, Gandara et al. developed a novel blood-based assay to calculate bTMB in NSCLC patients treated with atezolizumab. They identified cut-off points for bTMB using two randomized phase III trials, POPLAR and OAK, which included more than 1000 prospectively collected blood samples. Patients with bTMB ≥ 16 showed better clinical benefits from atezolizumab treatment than those with bTMB < 16. Interestingly, the combination of bTMB ≥ 16 with PD-L1 immunohistochemical staining (PD-L1 TC3 or IC3 positive) could identify those who benefited most from atezolizumab treatment (HR for PFS: 0.38, HR for OS 0.23) [85]. Therefore, bTMB is a feasible biomarker to predict clinical outcomes in patients with NSCLC, and it is necessary to confirm whether it is also applicable to other indications beyond NSCLC.

### 4.4. Exosomes

Exosomes are small extracellular vesicles released from various cells and contain bioactive substances, such as proteins and nucleic acids. Recently, it has been shown that PD-L1 can be delivered from tumor cells to other cells through exosomes. Therefore, exosomes can negatively impact anti-tumor immune responses. Notably, some studies have reported the relationship between circulating exosomes and the response to ICI therapy in patients with advanced cancers [86,87,107].

Chen et al. reported that PD-L1-expressing exosomes were released from melanoma cells and facilitated melanoma progression by suppressing CD8^+^ T cells. They showed that the expression levels of PD-L1 on exosomes distinguished melanoma patients from healthy donors. Moreover, lower baseline levels of exosomal PD-L1, but not of the total circulating PD-L1 levels, were associated with better clinical responses to pembrolizumab, and clinical responders displayed increases in exosomal PD-L1 within 6 weeks of treatment initiation. Intriguingly, the absolute level and fold changes of proliferating (Ki-67^+^) PD-1^+^ CD8 T cells were positively correlated with exosomal PD-L1 in the peripheral blood [86].

Another study showed the predictive role of exosomes in patients with advanced melanoma treated with ipilimumab. Patients with higher baseline PD-1 and CD28 expression on tumor-derived exosomes had longer PFS and OS than those with lower expression. Moreover, an increase in CD80 and CD86 molecules on DC-derived exosomes before and after treatment was correlated with the clinical response to ipilimumab [87].

Overall, circulating exosomes can act as important immune regulators during anti-cancer immune responses and have potential value as predictive biomarkers for ICI treatment.

## 5. Conclusions

Peripheral blood biomarkers are rapidly emerging in the field of immuno-oncology. They not only reflect tumor biology, but also provide in-depth information about the constantly changing host immune responses against the tumor. A number of translational studies have demonstrated the prognostic or predictive value of various blood biomarkers during ICI treatment. These results have allowed us to better understand the complex and dynamic interactions between cancer cells and the host immune system during ICI treatment, and have helped, at least in part, distinguish patients who will respond favorably to ICI treatment from those who will not. However, as studies to date have been mostly limited to lung cancer and melanoma, additional studies should be conducted to confirm whether this is reproducible in other cancer types. Since different cancers may have different immunosuppressive cells and cytokines within the TME, and different ICIs may have distinct immunological mechanisms of action, the specificity of biomarkers may differ depending on the type of cancer and ICI. Therefore, biomarker development and optimization for ICIs should be tailored to the immunological characteristics of each cancer type and drug, rather than using a “one-size-fits-all” approach. Moreover, since various multiplexed assays are employed for peripheral blood analyses, these assay protocols and their reporting methods need to be standardized, and additional studies will also be needed on the sampling timepoints, sensitivity, and specificity of each assay for clinical applicability. In prospective clinical trials, peripheral blood biomarkers need to be developed as companion diagnostics for ICIs to enable further optimization of cancer immunotherapy.

## Figures and Tables

**Table 1:** Summary of circulating immune cells associated with immunotherapy response.

Marker	Cancer Type	Treatment	N	Findings Associated with Clinical Response	Reference
CD8 T cells	NSCLC	PD-(L)1 inhibitor	94	Lower number of baseline CD8 T cells were associated with DCB and longer PFS (AUC 0.68 with combined modeling of pretreatment tumor PD-L1, bTMB, and circulating CD8 T cells).	Nabet et al., 2020 [18]
PD-L1^+^ CD8 T cells	Melanoma	Ipilimumab ±Nivolumab	190	Higher baseline levels of PD-L1^+^ CD8 T cells were associated with poor OS (AUC 0.76).	Jacquelot et al., 2017 [26]
PD-1^+^ CD8 T cells, NK cells	NSCLC	Nivolumab	31	Higher frequencies of PD-1^+^ CD8 T cells and active NK cells at baseline were associated with clinical benefit, PFS, and OS (AUC 0.85, 79% sensitivity, and 83% specificity).	Mazzaschi et al., 2019 [27]
PD-1^+^ CD8 T cells	MSI-high gastric cancer	Pembrolizumab	19	On-treatment increases in the frequencies of PD-1^+^ CD8 T cells were associated with DCB.	Kwon et al., 2021 [28]
Ki-67^+^ PD-1^+^ CD8 T cells	NSCLC	PD-1 inhibitor	29	Early proliferation (Ki-67^+^) of PD-1^+^ CD8 T cells within 4 weeks of treatment correlated with good response.	Kamphorst et al., 2018 [29]
Ki-67^+^ CD8 T cells	Melanoma	Pembrolizumab	47	Higher frequencies of Ki-67^+^ CD8 T cells to tumor burden at week 6 (> 1.94) were associated with better clinical outcomes.	Huang et al., 2017 [30]
Ki-67^+^ CD8 T cells	Thymic epithelial tumor/ NSCLC	Pembrolizumab or nivolumab	64/ 46	A fold change of Ki-67^+^ among PD-1^+^ CD8 T cells at baseline and day 7 (Ki-67 _D7/D0_) ≥2.8 correlated with DCB, PFS, and OS (AUC 0.89/0.81).	Kim et al., 2019 [31]
TIM-3^+^ T cells	NSCLC, RCC	PD-1 inhibitor	43	On-treatment increases in the frequencies of TIM-3-expressing CD4 or CD8 T cells were negatively associated with clinical responses and PFS. CD4^+^ T_CM_ cells at baseline were associated with good response and prognosis.	Julia et al., 2019 [32]
TIM-3^+^ T cells	Esophageal cancer	Nivolumab	20	On-treatment increases in the frequencies of TIM-3 expressing CD4 or CD8 T cells were correlated with better responses and OS.	Kato et al., 2018 [33]
Immunosenescent CD8 T cells	NSCLC	PD-(L)1 inhibitors	83	Higher percentage of pretreatment immunosenescent CD8 T cells (CD28^−^ CD57^+^ KLRG1^+^) was correlated with lower RR, DCB, worse PFS or OS (35% sensitivity, 100% specificity).	Ferrara et al., 2021 [34]
T_CM_/T_eff_ ratio	Melanoma NSCLC	Nivolumab	43/ 40	Higher pretreatment T_CM_/T_eff_ ratio was correlated with longer PFS.	Manjarrez-Orduno et al., 2018 [35]
CD8^+^ T_EM_ cells	Melanoma	PD-1 inhibitor	51	Higher frequency of CD8^+^ T_EM_ cells within 4 weeks of treatment initiation was correlated with the clinical benefit.	Krieg et al., 2018 [36]
CD8^+^ T_EM_ type 1 cells	Melanoma	Ipilimumab	137	High frequencies of CD8^+^ T_EM_ type 1 T cells (CD45RA^−^ CCR7^−^ CD27^+^ CD28^+^) at baseline were correlated with higher RR and longer OS.	Wistuba-Hamprecht et al., 2017 [37]
CD8^+^ T_EM_ cells, TIGIT^+^ PD-1^+^ CD8 T cells	NSCLC	PD-(L)1 inhibitors	263	Lower frequency of CD8^+^ T_EM_ cells and higher frequency of severely exhausted T cells (TIGIT^+^ PD-1^+^ CD8^+^) at baseline were associated with HPD and shorter OS.	Kim et al., 2019 [38]
TCR diversity and clonality of PD-1^+^ CD8 T cells	NSCLC	PD-(L)1 inhibitors	40	Pretreatment high PD-1^+^ CD8^+^ TCR diversity and increasing PD-1^+^ CD8^+^ TCR clonality after treatment were related to longer PFS and OS (87% sensitivity, 94% specificity).	Han et al., 2020 [39]
Highly differentiated CD4 T cells, Tregs	NSCLC	PD-(L)1 inhibitors	83	High proportion of highly differentiated CD4 T cells (CD27^−^ CD28 ^low/−^) and low percentage of CD25^+^ FOXP3^+^ CD4^+^ Tregs at baseline were associated with higher RR, longer PFS and OS (70% sensitivity, 100% specificity).	Zuazo et al., 2019 [40]
CD62 L^low^CD4^+^ T cells, Tregs	NSCLC	Nivolumab	126	Higher CD62L^low^ CD4^+^ T cell level at baseline was associated with higher RR, longer PFS, or OS. Conversely, the percentage of CD25^+^ FOXP3^+^ CD4^+^ Tregs was lower in responders (85.7% sensitivity, 100% specificity).	Kagamu et al., 2020 [41]
M-MDSC	Melanoma	Ipilimumab	68	Lower frequency of M-MDSCs (Lin^−^ CD14^+^ CD11b^+^ HLA-DR^low/−^) at baseline or at week 6 was related to ICI response and OS.	Kitano et al., 2014 [42]
M-MDSCs, Tregs	Melanoma	Ipilimumab	209	Pretreatment M-MDSCs (Lin^−^CD14^+^ HLA-DR^−/low^) were negatively correlated with OS, while Tregs (CD4^+^ CD25^+^ FoxP3^+^) were positively correlated with OS.	Martens et al., 2016 [43]
M-MDSCs, Tregs	Melanoma	Neoadjuvant ipilimumab	35	Early on-treatment decrease in M-MDSCs (Lin1^−^ HLA-DR^−^ CD33^+^ CD11b^+^) and increase in Tregs at 6 weeks were associated with longer PFS.	Tarhini et al., 2014 [44]
LOX-1^+^ PMN-MDSCs, Tregs	NSCLC	Nivolumab	63	High ratio of Treg to LOX-1^+^ PMN-MDSCs ≥ 0.39 after treatment was correlated with longer PFS (87.5% sensitivity, 72.2% specificity).	Kim et al., 2019 [45]

Non-small cell lung cancer, NSCLC; programmed death-ligand1, PD-(L)1; renal cell carcinoma, RCC; durable clinical benefit, DCB; overall survival, OS; progression-free survival, PFS; response rate, RR; central memory T cells, T_CM_; effector memory T cells, T_EM_; effector T cells, T_eff_; hyperprogressive disease, HPD; regulatory T cells, Tregs; myeloid-derived suppressor cells, MDSCs; monocytic-MDSCs, M-MDSCs; polymorphonuclear-MDSCs, PMN-MDSCs; microsatellite instability, MSI.

**Table 2:** Summary of cytokines and soluble factors associated with immunotherapy response.

Marker	Cancer Type	Treatment	N	Findings Associated with Clinical Response	Reference
IL-6	NSCLC	PD-(L)1 inhibitors	47	On-treatment decrease in IL-6 level was associated with improved PFS.	Keegan et al., 2020 [70]
IL-8	Melanoma, NSCLC	PD-1 inhibitors ± Ipilimumab	44 /19	On-treatment decrease in serum IL-8 level could be used to monitor and predict clinical benefit from ICIs (AUC 0.97 among three different patient groups).	Sanmamed et al., 2017 [72]
	UC, RCC	Atezolizumab	1445	High baseline levels of IL-8 were associated with decreased efficacy of atezolizumab. On-treatment decrease in IL-8 was correlated with improved OS.	Yuen et al., 2020 [71]
IL-10	Melanoma	Ipilimumab	35	Combination of IL-10 and TGF-β was associated with PFS.	Tarhini et al. 2015 [73]
	Melanoma	PD-1 inhibitors	18	Higher baseline IFN-γ/IL-10 ratio in PBMCs predicted longer PFS (AUC 0.96).	Giunta et al., 2020 [74]
Soluble CTLA-4	Melanoma	Ipilimumab	113	Higher serum levels of soluble CTLA-4 at baseline were associated with better ORR and OS.	Pistillo et al., 2018 [75]
Soluble PD-1/ PD-L1	Melanoma	PD-1 inhibitors	222	Elevated baseline serum PD-1 or PD-L1 levels predicted poor outcome (AUC 0.61 for sPD-L1 and 0.53 for sPD-1 in OS).	Ugurel et al., 2020 [76]
CRP	Various solid tumors	PD-1 inhibitors	326	Elevated baseline CRP was an indicator of poor RFS and OS.	Livanainen et al., 2019 [77]
	RCC	Nivolumab	42	The early on-treatment CRP flare-response was associated with tumor shrinkage and improved survival outcomes.	Fukuda et al., 2021 [78]
LDH	Various solid tumors	Immunotherapy	155	High baseline LDH levels were correlated with poor OS.	Bigot et al., 2017 [79]
	Melanoma	Pembrolizumab	616	Low pretreatment values of LDH were associated with favorable OS.	Weide et al., 2016 [80]
CTCs	NSCLC	PD-(L)1 inhibitors	104	The presence of CTC is a predictive factor for a worse durable response rate to ICI.	Tamminga et al., 2019 [81]
ctDNA	NSCLC	Durvalumab	28	A drop in the ctDNA level is an early marker of therapeutic efficacy and predicts prolonged survival in patients treated with ICIs.	Raja et al., 2018 [82]
	NSCLC	Pembrolizumab + chemotherapy	62	Decreases in ctDNA levels were related with clinical benefit.	Ricciuti et al., 2021 [83]
	Various solid tumors	Pembrolizumab	94	Baseline ctDNA levels were correlated with PFS, OS, and clinical response.	Bratman et al., 2020 [84]
bTMB	NSCLC	PD-(L)1 inhibitors	98	ctDNA-based bTMB could be used as a potential biomarker for anti-PD-1 and anti-PD-L1 treatment in patients with NSCLC.	Wang et al., 2019 [60]
	NSCLC	Atezolizumab	216	High bTMB is a clinically actionable biomarker for atezolizumab.	Gandara et al., 2018 [85]
Exosome	Melanoma	Pembrolizumab	44	Lower baseline levels of exosomal PD-L1 and their increase during treatment were correlated with tumor response (AUC 0.91 for exosomal PD-L1, 0.70 for total PD-L1).	Chen et al., 2018 [86]
	Melanoma	Ipilimumab	59	Increased exosomal PD-1, and the CD28 levels in T cells were associated with longer PFS and OS.	Tucci et al., 2018 [87]
